# An Almoner's Work in a General Hospital

**Published:** 1907-05-04

**Authors:** 


### AN ALMONER'S WORK IN A GENERAL HOSPITAL.

The work of an almoner in the out-patient department of a hospital can, after ten years' trial, no longer be considered experimental. From the selection of a few cases for inquiry it has developed into a systematic interrogation of practically all the out-patients, as far as possible after their first treatment. Has the work justified the hopes of those who saw in it a means of lessening the abuse of the out-patient department, with its problems of overcrowding and ever increasing financial burden; or has it fulfilled the fears of those who thought the introduction of such a system would ruin the out-patient department for clinical purposes?

The abuse of a voluntary hospital by the well-to-do, who can afford to pay a private practitioner, is undoubtedly checked by careful inquiry into the circumstances of each case, but the proportion of such cases in a hospital in a poor neighbourhood is very small, and tends to become less, for the fact that inquiry is made is in itself a deterrent.

### The Wider Aspects of the Problem.

If, however, in the term "abuse" be included the "waste" of much out-patient treatment, the problem assumes far larger proportions.

1. The waste by the ignorant, whose insanitary conditions at home, and whose want of knowledge of the simplest rules of health outweigh any good that medical treatment might do. By an intimation of the home conditions to the medical officer, and the introduction of a health visitor, district nurse, or suitable worker, the almoner may do much to create a feeling that hospital treatment is a privilege only to be granted if the recipient is prepared to do his or her part towards the desired result.

2. The waste of inadequate help, where through some temporary distress, possibly the illness in question, the patient is unable to get the necessary rest, good food, or freedom from anxiety which alone can render the medical treatment of any real value. In co-operation with one of the many charitable agencies outside the hospital, the almoner will in many cases be able to arrange the additional help, the Samaritan Fund bearing part of the cost incurred.

3. The waste of treating the destitute whose needs can only be adequately met by the Poor-law authorities. A closer co-operation between Poor-law officers and hospital authorities, based upon a clearer conception of the functions of voluntary hospitals and Poor-law infirmaries, would seem a mutual advantage; but so far as out-patients are concerned the almoner's course is clearly to refer all destitute patients, or those dependent upon the Poor-law, to the parish doctor, who, of course, has the right to send any patient to hospital for a special opinion or for admission.

4. The waste of treating minor ailments for which, if they need treatment at all, provision can easily be made outside the hospital. The systematic discouragement of such cases and their reference to private practitioners, provident dispensaries, or friendly societies, seems the only course open to the almoner. So long as there is no uniform system in hospitals and very little interchange of information between them, it is feared that this often means their attending another hospital. A uniform system, with some distance limit for patients, would do much to prevent this over-lapping.

It will doubtless be asked how the inquiry which is to determine the course of action in either of these cases is conducted, and whether experience shows that any reliance can be placed upon statements which are not verified?

### The Qualifications of the Ideal Almoner.

An almoner who hopes for any measure of success must start her work with a thoroughly practical knowledge of the social conditions and habits of the people among whom she is going to work, of the agencies which exist to improve their condition, and of the facilities to enable them to provide against the contingencies of life. Having been accustomed to receive and formulate their statements, and to test the accuracy by inquiry, she will quickly realise that if a wage limit be imposed, or if the questions asked be stereotyped, false statement will surely be the result.

The object of the inquiry, which should be more or less of the nature of a friendly talk, being to get a general idea of the position of the patient, to get behind his statement, more importance will attach to the nature of his work, the length of his jobs, the kind of work he has chosen for his children, or to his practice in sickness, than to the actual amount of his last week's earnings; while in the case of a man who is out of work, important factors are the nature of his trade, whether seasonal or otherwise, the length of his tenancy at his various addresses, what friends he has behind him, and whether he has been obliged to apply to the Guardians.

The almoner trained by the Charity Organisation Society has access to the records of that Society, the use of its machinery for inquiry, as well as the valuable help of its experienced visitors all over London; and her own position in the out-patient department will give her opportunity of knowing and of being helpful to the clergy, relieving officers, and the many social workers in her own district, who, from time to time, want information about patients; and they in their turn will be ready to help her. As a written record is kept of every case in which any action is taken, together with any information obtained, opportunity is afforded of comparing and correcting the original statement made by the patient, and experience shows that deliberate deception is not frequent. The practice of encouraging patients to repay the cost of their appliances, etc., in instalments has been found useful, not only as encouraging self-help, but as a means of knowing the family. The weekly visit for payments creates a pleasantly independent relationship, and frequently results in confidences which a formal inquiry has failed to elicit.

### The Value of the Work.

The constant demands made upon the almoner justify the conclusion that the work is found useful by the medical staff, while the numerous cases which might be quoted where restoration to health, recovery of independence, of work under suitable conditions, have been the result of concerted action, show that thoughtful and well-considered charity is both economic and constructive. As regards numbers, the fact that the special departments tend to increase is satisfactory, in so far as they provide treatment which comparatively few of our patients can obtain for themselves, while in these departments is found the material for clinical purposes. The steady decrease of minor cases treated in the out-patient department would also be encouraging were it not that very many such cases obtain treatment in the accident department on the plea of emergency.

In this department, which is open day and night, there is no classification of patients, so that the responsibility of regulating it must rest with those who are medically qualified. Suitable patients are referred to the out-patient department, if a second attendance is necessary; but even so, the dressing of cuts and bruises, and the dosing of all comers is a costly and ineffective form of charity, destructive of thrift and foresight, and the usefulness of a hospital can in no sense be estimated by the thousands so treated.

